# Spontaneous twin pregnancy with live births after cryopreservation and re-implantation of ovarian tissue

**DOI:** 10.1186/s10397-017-1012-6

**Published:** 2017-06-14

**Authors:** Milan Milenkovic, Mats Brännström, Cesar Diaz-Garcia, Kersti Lundin, Ulrika Selleskog, Brita Söderlund, Ali Khatibi, Berit Gull, Hans Bokström, Claudia Mateoiu, Levent M. Akyürek, Ann Thurin-Kjellberg

**Affiliations:** 1 Department of Obstetrics and Gynecology, Sahlgrenska Academy Hospital, University of Gothenburg, Gothenburg, Sweden; 2 Department of Obstetrics and Gynecology, La Fe University Hospital, University of Valencia, Valencia, Spain; 3 Human Reproduction Research Group, IIS La Fe, Valencia, Spain; 4 Department of Clinical Pathology and Genetics, Sahlgrenska Academy Hospital, University of Gothenburg, Gothenburg, Sweden; 5 Department of Obstetrics and Gynecology, Karolinska University Hospital, Stockholm, Sweden

**Keywords:** Fertility preservation, Ovarian transplantation, Pregnancy, Twin, Livebirth

## Background

Ovarian tissue cryopreservation (OTC) before gonadotoxic treatment and subsequent avascular auto-transplantation is a method of fertility preservation in cancer patients, with first birth reported in 2004 [1]. This method has resulted in more than 85 livebirths worldwide [2–4], with delivery rate of 25–32% per transplanted woman [2, 3].

## Methods

**Fig. 1:**
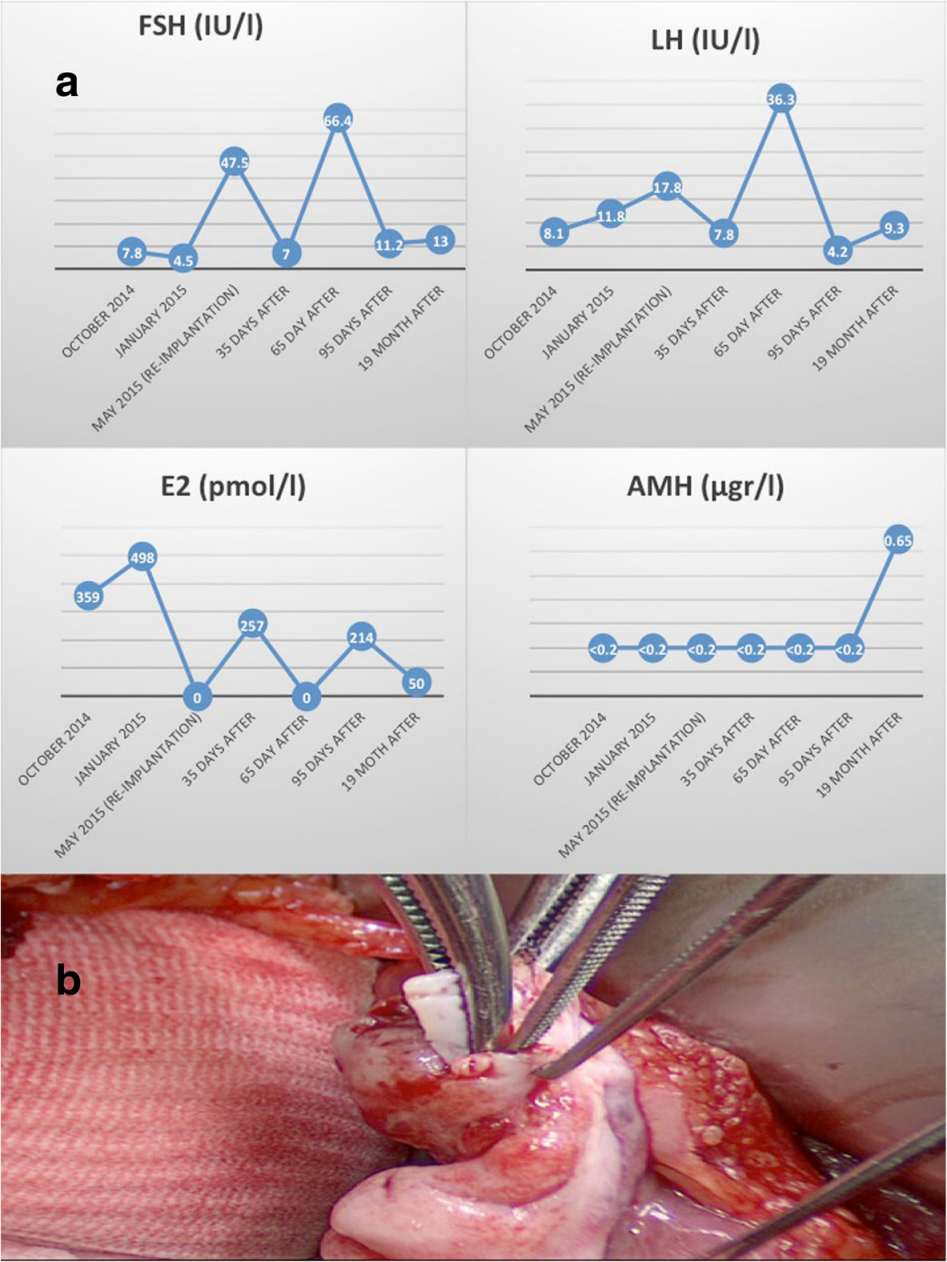
**a** Hormonal status after chemotherapy (follicle stimulating hormone (FSH); luteinising hormone (LH); estradiol (E2); anti-Müllerian hormone (AMH)). **b** Re-implantation of thawed ovarian tissue in subcortical pocket of the left ovary

A 27-year-old, 1-parous patient suffered from Hodgkin’s lymphoma in 2011. Pre-operative transvaginal sonography (TVS) revealed a normal uterus, the left side ovary with a unilocular cyst of 50 × 70 mm and the right ovary with 8 antral follicles. Laparoscopic stripping of left ovarian cyst and right-sided oophorectomy was performed with subsequent standard OTC of 15 cortical strips [5] before six chemotherapy treatments with BEACOPP (bleomicin, etoposide, adryamicin, cyclophosphamide, oncovin, procarbazine, prednisolone). Histology showed benign mucinous cystadenoma. After chemotherapy, patient experienced amenorrhea and climacteric symptoms that were treated by hormonal replacement therapy (HRT). HRT was ceased in October 2013 due to benign cysts in the breast, and the patient developed oligomenorrhea. Hormonal status after chemotherapy is presented in Fig. 1a. Patient was considered free of disease and tried to conceive for 1 year. In March 2015, TVS showed cyst on the left ovary and laparoscopic excision of the cyst and cortex biopsy was performed. Histology of the cortex showed 0.2 primordial follicles (PF)/mm^2^ and cyst without atypia. Patient consented to re-implant cryopreserved ovarian tissue. Tissue was thawed, and histology demonstrated 8–10 PF/mm^2^ and no atypical cells. A mini-laparotomy through Pfannenstiehl incision and re-implantation of thawed ovarian tissue was performed in May 2015. Four pieces were re-implanted in subcortical pockets of the left ovary (Fig. 1b) [2], and due to the small remaining ovary, three pieces were placed under the peritoneum of the right mesosalpinx [1]. On the day of cortex re-implantation, hormonal status confirmed ovarian failure (Fig. 1a). TVS showed no antral follicles.

## Findings

Menstrual bleedings occurred 21, 40, and 51 days after re-implantation. Thirty days after the surgery, TVS demonstrated corpus luteum on the left ovary, and the transplant measured 15 × 8 × 11 mm on the right side. Progesterone level was 28 nmol/l. TVS on day 112 after re-implantation demonstrated triple endometrium on 7 mm, two antral follicles on the left ovary, and one follicle on the right side. One hundred thirty-four days after re-implantation, TVS confirmed spontaneous viable dichoriotic diamniotic twin intrauterine pregnancy corresponding to pregnancy week 5 + 4. Spontaneous vaginal delivery occurred at gestational week 37 + 2 of a healthy girl and boy weighing 2085 and 2480 g, respectively. Nineteen months after transplantation, the patient has regular menstrual bleeding.

## Discussion

To our best knowledge, we report a first spontaneous twin pregnancy after OTC and auto-transplantation. Twin pregnancies and livebirths following OTC and IVF were published before [4]. Estradiol production by ovarian cyst can explain normal gonadotropin level 4 months before re-implantation. Gonadotropins increased to castrated level after cyst excision (Fig. 1a). We cannot completely rule out the possibility that early bleeding pattern reflected hormonal activity of the native ovary. However, pregnancy was already reported 2 months after ovarian tissue transplantation [3]. Regular menstrual pattern after delivery confirms graft hormonal function. Re-implantation may be performed by laparoscopy or by laparotomy [1–4]. We choose surgery by 6-cm laparotomy using surgical loops and respecting microsurgical rules, after our experience on uterus transplantation and animal experiments [6, 7]. Otherwise, this is the first auto-transplantation performed at Sahlgrenska Academy Hospital since the OTC program was introduced in 1995.
